# Necrology

**Published:** 1882-03

**Authors:** 


### Necrology.

---

DR. CASS MASON DODGE, died at his office, corner Halsted street and Archer avenue, of diphtheria, December 20, 1881. Dr. Dodge was born at Prospect Park, Ill., in 1848, he graduated at Rush Medical College, in 1873, and practiced in Chicago for several years. He leaves a wife and two children. He contracted his last sickness from one of his patients, a peril to which each physician is greatly exposed, though the fact is never taken into account by the community.

DR. JOSIAH C. CHURCH, died February 13, 1882, at the residence of his son, Dr. Nelson H. Church, No. 17 Filmore street. Dr. Church was born in New York State, in 1815, received the honorary degree of M. D. from Syracuse University. He practiced many years in Geneva, N.Y., and twenty-two years in Patoka, Ind. Paralysis, the disease of which he died, had confined him to his bed for four years.

DR. JAMES ALLEN MEAD, died at his office, 54 S. Canal street, of typhoid fever, February 16, 1882. He was born in New York State, in 1841, and kept a drug store for sixteen years. He studied medicine in Rush Medical College, and received his degree in 1876. The few last years of his life were confined to practice. Dr. Mead had been rather successful in pecuniary matters, leaving a small fortune to his wife and two young children.

DR. DANIEL S. ROOT, died at his office, corner of Twelfth and Halsted streets, February 23, 1882, of chronic diarrhœa, probably tubercular. Dr. Root graduated in Rush Medical College, in 1867, and founded a large practice which proved very lucrative. He remained single, and died aged about forty.

